# Differential Activation of the Wheat SnRK2 Family by Abiotic Stresses

**DOI:** 10.3389/fpls.2016.00420

**Published:** 2016-03-31

**Authors:** Hongying Zhang, Weiyu Li, Xinguo Mao, Ruilian Jing, Hongfang Jia

**Affiliations:** ^1^Key Laboratory for Cultivation of Tobacco Industry, College of Tobacco Science, Henan Agricultural UniversityZhengzhou, China; ^2^College of Plant Science and Technology, Key Laboratory of New Technology in Agricultural Application, Beijing University of AgricultureBeijing, China; ^3^The National Key Facility for Crop Gene Resources and Genetic Improvement, Institute of Crop Science – Chinese Academy of Agricultural SciencesBeijing, China

**Keywords:** *TaSnRK2s*, abiotic stress, ABA signaling, gene expression, protein–protein interaction, *Triticum aestivum*

## Abstract

Plant responses to stress occur via abscisic acid (ABA) dependent or independent pathways. Sucrose non-fermenting1-related protein kinase 2 (SnRK2) play a key role in plant stress signal transduction pathways. It is known that some SnRK2 members are positive regulators of ABA signal transduction through interaction with group A type 2C protein phosphatases (PP2Cs). Here, 10 *SnRK2s* were isolated from wheat. Based on phylogenetic analysis using kinase domains or the C-terminus, the 10 *SnRK2s* were divided into three subclasses. Expression pattern analysis revealed that all *TaSnRK2s* were involved in the responses to PEG, NaCl, and cold stress. *TaSnRK2s* in subclass III were strongly induced by ABA. Subclass II *TaSnRK2s* responded weakly to ABA, whereas *TaSnRK2s* in subclass I were not activated by ABA treatment. Motif scanning in the C-terminus indicated that motifs 4 and 5 in the C-terminus were unique to subclass III. We further demonstrate the physical and functional interaction between TaSnRK2s and a typical group A PP2C (TaABI1) using Y2H and BiFC assays. The results showed that TaABI1 interacted physically with subclass III TaSnRK2s, while having no interaction with subclasses I and II TaSnRK2s. Together, these findings indicated that subclass III TaSnRK2s were involved in ABA regulated stress responses, whereas subclasses I and II TaSnRK2s responded to various abiotic stressors in an ABA-independent manner.

## Introduction

Abscisic acid (ABA) is an essential plant hormone that is involved in the abiotic stress response. As a stress hormone, ABA acts through regulatory pathways that control gene expression and stomatal closure (Zhu, 2002; Wasilewska et al., 2008; Lee and Luan, 2012). Increasing evidence shows that the PYR/PYL/RCARs-PP2C-SnRK2 pathway is an essential ABA-dependent stress-signaling pathway and plays a crucial role in the abiotic stress response. There are three major components of the PYR/PYL/RCARs-PP2C-SnRK2 pathway: proteins conferring pyrabactin resistance (1/PYR1-like/regulatory proteins) include ABA receptors (PYR/PYL/RCAR), group-A protein phosphatases 2C (PP2C) and sucrose non-fermenting-1 related protein kinase 2 (SnRK2). In the absence of ABA, PP2C inhibits SnRK2 by direct dephosphorylation. In response to environmental stress, ABA accumulates in plant cells. Increasing endogenous ABA can be sensed and bound by the PYR/PYL/RCAR proteins and repress the activity of PP2C that would otherwise inhibit SnRK2; without PP2c activity, SnRK2 proteins become phosphorylated and trigger the expression of ABA-responsive genes in an ABRE-dependent manner (ABA responsive pathway; Ma et al., 2009; Melcher et al., 2009; Nishimura et al., 2009; Park et al., 2009; Sheard and Zheng, 2009). In this context, group A PP2Cs (e.g., ABI1 and ABI2), function as negative regulators of the ABA response, while SnRK2s act as positive regulators in the ABA signaling pathway (Merlot et al., 2001; Saez et al., 2004; Kuhn et al., 2006; Hirayama and Shinozaki, 2007; Rubio et al., 2009; Nishimura et al., 2010).

The SnRK2s are a relatively small plant-specific gene family, encoding serine/threonine kinases. Increasing evidence shows that individual SnRK2 members have acquired distinct regulatory properties in the abiotic stress response, including ABA responsiveness. In general, SnRK2s in subclass III are strongly activated by ABA, while subclass II members are weakly induced by ABA. Subclass I induction by ABA are moderate (Kobayashi et al., 2004, 2005; Boudsocq et al., 2007; Huai et al., 2008; Fujita et al., 2009). In *Arabidopsis*, 10 group A PP2Cs interact with SnRK2b members were tested by the yeast two-hybrid (Y2H) assay and consistent with previous studies, SnRK2 subclass III members interacted strongly with group A PP2Cs in various combinations, while subclass II members showed limited interaction with group A PP2Cs (Umezawa et al., 2009). Increasing evidence shows that ABI, a typical member of group A PP2Cs, interacts with subclass III SnRK2s in ABA in the signal transduction pathway (Yoshida et al., 2006; Fujita et al., 2009; Umezawa et al., 2009). Until now, there has been no specific evidence demonstrating whether PP2C interacts with individual SnRK2 members directly.

In wheat, additional SnRK2 members have been reported. The first member of the wheat SnRK2 family, *PKABA1*, was cloned from an ABA-treated wheat embryo cDNA library (Anderberg and Walker-Simmons, 1992). Thereafter, four *PKABA1*-like protein kinase genes (*TaPK3*, *W55a*, *W55b*, *W55c*, and *TaSRK2C1*) were identified from wheat. The results revealed that all the genes were activated by multiple stressors or ABA application (Holappa and Walker-Simmons, 1997; Xu et al., 2009; Du et al., 2013). In our recent study, four wheat SnRK2 genes (*TaSnRK2.3*, *TaSnRK2.4*, *TaSnRK2.7*, *TaSnRK*2.8) were cloned. All four genes responded to multiple stressors, though through different mechanisms including ABA responsiveness. Among them, *TaSnRK*2.*3* and *TaSnRK2.8* were upregulated by ABA treatment, whereas *TaSnRK*2.3 and *TaSnRK2.7* were not regulated by ABA (Mao et al., 2009; Zhang et al., 2010, 2011; Tian et al., 2013). Other wheat SnRK2 members have been identified through searches of the National Center for Biotechnology Information (NCBI) databases, although their specific functions have not been characterized. To date, little is known about the function of wheat SnRK2 members in ABA signaling that activates gene expression in response to abiotic stress.

In this study, we isolated 10 SnRK2 members from wheat and characterized their expression patterns under abiotic stress and ABA treatments. To better understand the function of wheat SnRK2 kinases in ABA signaling, we analyzed the promoter sequences and conserved motifs of *SnRK2s*, and further demonstrated the biochemical relationship between PP2C and SnRK2 using co-immunoprecipitation, Y2H and bimolecular fluorescence complementation BiFC assays.

## Materials and Methods

### Cloning of Wheat SnRK2 Members and Sequence Analysis

Based on the sequence data of *SAPK5* (AB125306), *SAPK6* (AB125307), *SAPK9* (AB125310), and *SAPK10* (AB125311), the putative full-length cDNAs of *TaSnRK2.5*, *TaSnRK2.6*, *TaSnRK2.9*, and *TaSnRK2.10* were inferred by *in silico* cloning. To obtain the full-length cDNAs, primers were designed from sequences flanking the ORF of the putative sequence (**Supplementary Table S1**). The full-length cDNAs of *TaSnRK2.3*, *TaSnRK2.4*, *TaSnRK2.7*, and *TaSnRK2.8* were obtained from our previous researches (Mao et al., 2009; Zhang et al., 2010, 2011; Tian et al., 2013). Sequences data of *TaSnRK2.1* (TC368696), *TaSnRK2.2* (KF688097), and *TaSnRK2.10* (KJ0187223) were obtained from NCBI databases.

C-terminal conserved motifs of *TaSnRK2s* were determined using software tools available at MEME^1^.

To determine the relationships between *TaSnRK2s* and SnRK2 homologs in other plant species, CLUSTAL W (1.82) and PHYLIP software (version 3.69) were used to construct a phylogenetic tree, which was viewed using TREEVIEW software.

### Promoter Analysis

To isolate the promoter of *TaSnRK2s*, genomic sequences were used as queries to screen the wheat genome sequencing database using the newly developed rapid ENA sequence similarity search service^2^ and a genome sequence database of *A. tauschii* (DD; unpublished data), the diploid D genome donor species of common wheat. A significant match was declared when the queried sequences showed at least 95% nucleotide identity with an expectation value *e* = 0. The 2-kb region upstream of the translation start site of each gene was considered to be the putative promoter region. The abiotic stress-associated regulatory elements were predicted using the database of plant *cis*-acting regulatory DNA elements at the National Institute of Agrobiological Sciences^3^.

### Plant Treatment

Germinated seeds of hexaploid wheat (*Triticum aestivum* L., cv. “Hanxuan 10”) were cultured in a growth chamber (20°C, 12 h:12 h photoperiod). Seedlings at the two-leaf stage (9-days-old) were separately stressed by exposure to salt (250 mM NaCl solution); osmotic shock simulating water stress (PEG-6000 (-0.5 MPa) solution); low temperature (4°C) or direct application of ABA (50 μM ABA spray, which was shown to constitute a significant stress in pilot experiments). Untreated control seedlings were cultured normally. Wheat leaves were sampled at 0, 1, 3, 6, 12, 24, 48, and 72 h after treatments. RNA from each time point was extracted with TRIzol reagent (Invitrogen, Carlsbad, CA, USA). To remove traces of genomic DNA contamination, RNA samples were treated with DNase (Invitrogen).

### Expression Patterns of *TaSnRK2s*

Quantitative real-time PCR (qRT-PCR) was used to estimate *TaSnRK2s* transcript levels resulting from abiotic stresses and ABA application. qRT-PCR was performed with SYBR *Premix Ex Taq* (Takara, Shiga, Japan) using an ABI PRISM 7000 system (Applied Biosystems, Foster City, CA, USA). Specific primers were designed based on cDNA sequences (**Supplementary Table S2**). Expression of the wheat *tubulin* gene was used as an internal control for expression levels. The relative expression level of *TaSnRK2s* was calculated using the 2^-ΔΔCT^ method (Livaka and Schmittgen, 2001).

### Yeast Two-Hybrid Analysis

Yeast two-hybrid analysis was performed using the MatchMaker GAL4 two-hybrid system-3 (Clontech, USA) according to kit instructions. Yeast strain (AH109) was transformed with pGBKT7 vectors harboring the coding region of each *TaSnRK2* insert and a pGADT7 vector harboring the coding region of *TaABI1*. The transformants were grown on auxotrophic media as indicated in **Figure 4A**. Primers used for cloning are listed in **Supplementary Table S1**.

### Transient Expression Assay

The vectors pUC-SPYNE (yellow fluorescent protein [YFP] N-terminal fragment, YN) and, pUC-SPYCE (YFP C-terminal Fragment, YC), used in the bimolecular fluorescence complementation (BiFC) assay, were obtained from Harter and Kudla (Walter et al., 2004). The coding sequences of *TaSnRK2s* were cloned into pUC-SPYCE to create fusion constructs with the N-terminal fragment of yellow fluorescent protein (YN). The coding sequence of *TaABI1* was cloned into pUC-SPYNE to create a fusion with the C-terminal fragment of YFP (YC). The cloning primers are listed in **Supplementary Table S1**. The recombinant constructs were co-transfected into *A. tumefaciens* (strain GV3101), and then transferred into tobacco leaves by infiltration using a 1-mL syringe. For microscopic analyses, leaf disks were removed 4 days after infiltration. Cells of the lower epidermis were examined for YFP fluorescence with a laser scanning confocal microscope (Leika TCS-NT). Negative controls consisted of pUC-SPYNE co-transfected with pUC-SPYCE, or either blank vector paired with one of the treatment vectors.

Protein assays, immunoprecipitation and western blotting were performed as described previously (Umezawa et al., 2009). For immunoprecipitation experiments, transfected tobacco leaves were extracted in PBS containing 0.05% Triton X-100, 1 mM EDTA, and protease inhibitor cocktail (Roche). HA-tagged proteins were immunoprecipitated and purified using superparamagnetic micro MACS beads coupled to monoclonal anti HA antibody according to the manufacturer’s instructions (Sigma). Purified immunocomplexes were analyzed by immunoblotting using anti-myc antibody (Roche).

## Results

### Molecular Characterization of *TaSnRK2s*

Ten SnRK2 genes were identified from wheat (**Table 1**), which were designated *TaSnRK2.1* to *TaSnRK2.10*. Scansite analysis indicated that *TaSnRK2s* have potential serine/threonine protein kinases activities, and like other SnRK2s, contain an N-terminal catalytic domain and a C-terminal regulatory region. The N-terminal catalytic domain is highly conserved, containing an ATP binding site and protein kinase activating signature. The C-terminal regions of *TaSnRK2*s are quite divergent and contain regions rich in acidic amino acids.

**Table 1:** Summary information for *TaSnRK2* genes.

Name	ORF (bp)	Length (aa)	Mol. wt. (kd)	pI
*TaSnRK2.1*	1029	343	38.85	5.83
*TaSnRK2.2*	1026	342	38.64	5.51
*TaSnRK2.3*	1029	343	38.48	5.69
*TaSnRK2.4*	1092	364	42.15	6.05
*TaSnRK2.5*	969	323	37.02	10.35
*TaSnRK2.6*	1074	358	40.90	5.60
*TaSnRK2.7*	1074	358	40.91	5.61
*TaSnRK2.8*	1101	367	41.59	4.77
*TaSnRK2.9*	1047	349	38.94	5.03
*TaSnRK2.10*	1053	351	39.58	4.68


*Accession numbers of *TaSnRK2.1*, *TaSnRK2.2*, and *TaSnRK2.10* are TC368696, KF688097, and KJ0187223. The cDNAs of *TaSnRK2.3*, *TaSnRK2.4*, *TaSnRK2.7*, and *TaSnRK2.8* were obtained in our previous research (Mao et al., 2009; Zhang et al., 2010, 2011; Tian et al., 2013). Others are from the experiments of the present study.*

### Phylogenetic Analysis of TaSnRK2s Proteins

**FIGURE 1:**
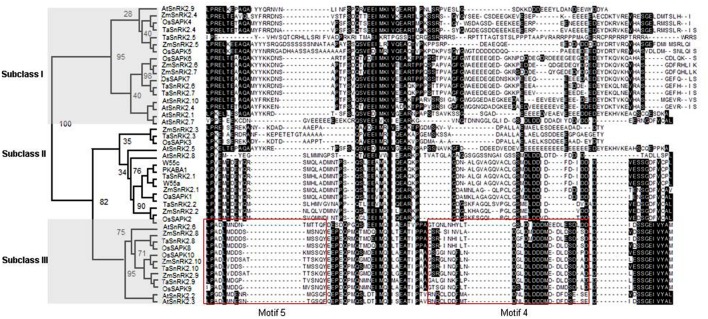
**Phylogenetic tree and sequence alignment of the C-terminus of SnRK2 family.** Sequences showed in the boxes are motifs 4 and 5 of subclass III TaSnRK2s. The phylogenetic tree was constructed putative amino acid sequences using the PHYLIP 3.68 package. Bootstrap values are in percentages. Os, *O. sativa*; At, *A. thaliana*; Zm, *Z. mays*; Ta, *T. aestivum*.

A phylogenetic tree was constructed with the amino acid sequences of the C-terminus of SnRK2 family proteins, including those from wheat, rice, maize, and *Arabidopsis*. Phylogenetic analysis indicated that these protein kinases were divided into three subclasses, namely subclasses I, II, and III, which contained TaSnRK2.4 through TaSnRK2.7 (Class I), TaSnRK2.1 through TaSnRK2.3 (Class II), and TaSnRK2.8 through TaSnRK2.10 (class III), respectively (**Figure 1**).

Based on functional and sequence divergence, SnRK2s are divided into three distinct subclasses, namely SnRK2a (corresponding to subclass I) and SnRK2b (corresponding to subclasses II and III; Halford and Hardie, 1998). Similar to previous reports, a comparison of C-terminal sequences suggested that there were more structural similarities between subclasses II and III than other pairs (**Figure 1**). For example, the C-terminal blocks in subclasses II and III are conserved; there are fewer amino acids in subclasses II and III than in subclass I; and the acidic patches of subclass I are abundant in Glu, while those of subclasses II and III are rich in Asp.

### Conserved Motifs in the C-terminus of TaSnRK2 Kinases

**FIGURE 2:**
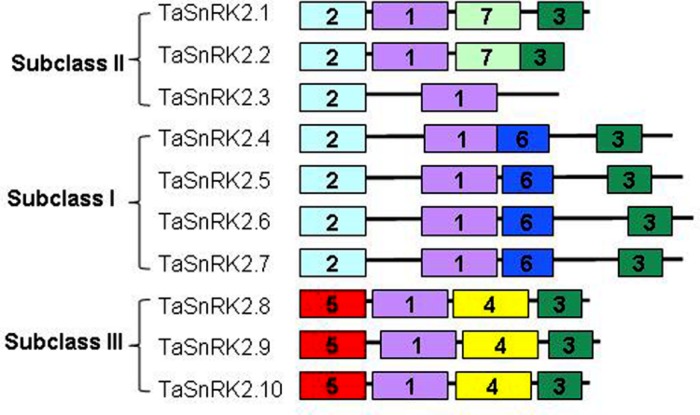
**Distribution of the seven conserved motifs in the C-terminus of TaSnRK2s.** Each motif is represented by a different colored box. Black lines represent the non-conserved sequences.

Increasing evidence has shown that there are seven functional domains in the C-terminal regions of SnRK2s although they are divergent (Yoshida et al., 2006; Yuasa et al., 2007; Huai et al., 2008). As shown in **Figure 2**, motif 1 existed in all TaSnRK2 members. Motif 3 was found in all SnRK2 members except TaSnRK2.3. Motif 2 is unique to subclasses II and III TaSnRK2s. Motifs 4 and 5 are unique to subclass III TaSnRK2s, and motif 6 was only identified in subclass I TaSnRK2s. Motif 7 is unique to TaSnRK2.1 and TaSnRK2.2.

### Regulatory Elements in the Promoters of *TaSnRK2s*

As shown in **Table 2**, 15 *cis*-acting elements related to stress responses in the promoter regions of the *TaSnRK2s* were identified, including ABA response elements (ABRE), low temperature response elements (LTREs), dehydration-response elements (ACGTATERD1 and DRE), and binding sites for MYB/MYC transcription factors. Nine MYB/MYC binding sites, which are related to biotic and abotic stress resistance (Du et al., 2012; Hou et al., 2012), were found in the promoters of *TaSnRK2s*. *TaSnRK2* members (1–10) had 27, 29, 28, 45, 47, 42, 40, 39, 29, and 26 copies of these elements, respectively. MYB/MYC binding sites were most common in subclass I *TaSnRK2s*. In addition, the numbers of ABRE elements existing in *TaSnRK2s* promoters (1–10) were 14, 2, 15, 1, 2, 13, 11, 2, 4, and 7. The subclass I *TaSnRK2s* (*TaSnRK2.4-7*), which were not activated by ABA, contain several ABREs in their promoter regions.

**Table 2:** Stress-related *cis* elements identified in the 2-kb upstream region of *TaSnRK2s*.

*Cis*-elemnent	Sequence	Code	*TaSnRK2* member									
			
			1	2	3	4	5	6	7	8	9	10
ABRELATERD1	ACGTG	S000414	7	2	9	0	2	7	6	1	2	5
ABRERATCAL	MACGYGB	S000507	7	0	6	1	0	6	5	1	2	2
ACGTATERD1	ACGT	S000415	12	8	18	2	4	16	16	4	8	10
DRECRTCOREAT	RCCGAC	S000418	0	3	1	3	2	1	2	1	3	6
LTRECOREATCOR15	CCGAC	S000153	0	4	1	4	2	4	3	1	4	6
LTRE1HVBLT49	CCGAAA	S000250	4	2	1	0	0	0	0	2	3	1
MYB1AT	WAACCA	S000408	3	2	4	4	0	0	3	4	0	3
MYBATRD22	CTAACCA	S000175	1	0	0	1	0	0	0	0	0	0
MYBCORE	CNGTTR	S000176	4	4	3	6	4	12	7	5	4	4
MYB2CONSENSUSAT	YAACKG	S000409	0	1	1	5	2	7	3	4	2	0
MYCCONSENSUSAT	CANNTG	S000407	14	12	14	18	34	12	14	14	18	16
MYBCOREATCYCB1	AACGG	S000502	2	2	0	5	1	6	5	7	2	1
MYBGAHV	TAACAAA	S000181	0	1	1	0	0	0	2	2	0	0
MYBPZM	CCWACC	S000179	2	4	2	3	2	2	2	1	2	1
MYBST1	GGATA	S000180	1	3	3	3	4	3	4	2	1	1


### Expression Patterns of SnRK2 Members of Wheat as a Function of Stressor

**FIGURE 3:**
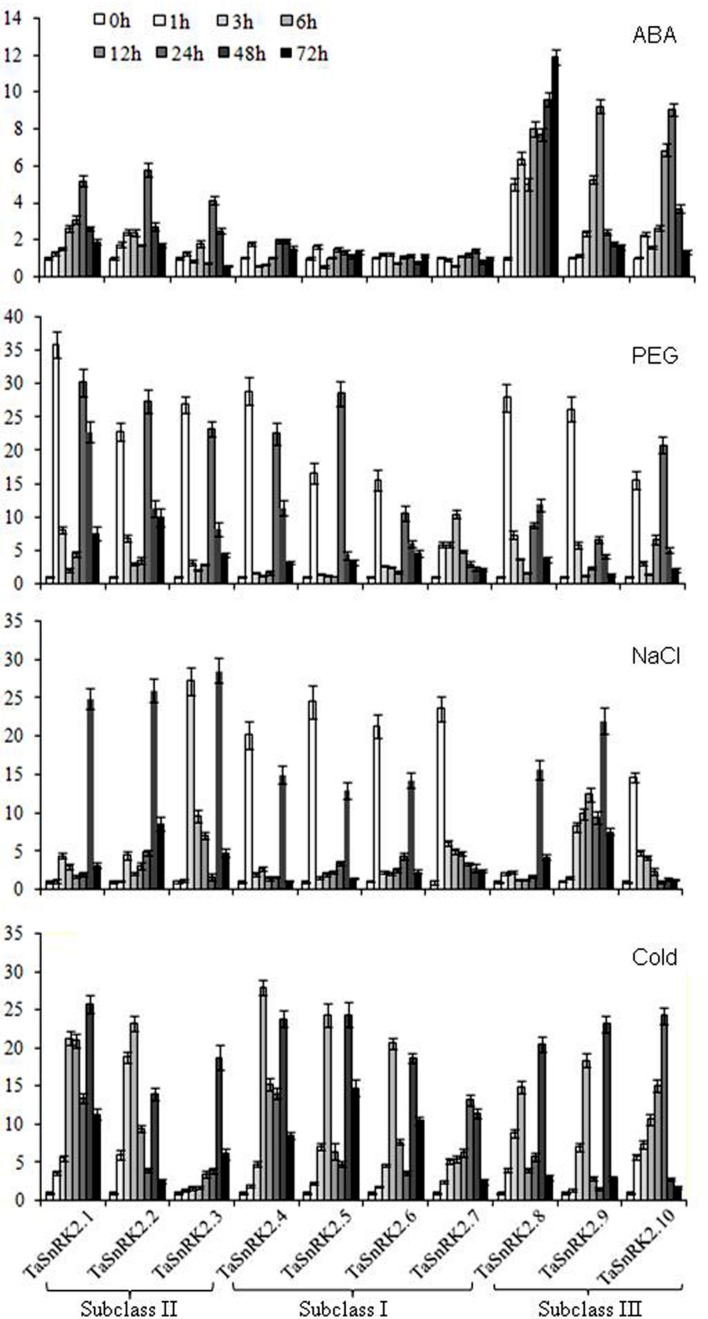
**Expression patterns of *TaSnRK2s* in response to ABA, PEG, NaCl, and cold treatments.** *Tubulin* was used as an internal control. The expression of *TaSnRK2s* at 0 h was regarded as the standard. Values are mean ± SD (*n* = 3).

The expression of the 10 *TaSnRK2*s was detected by qRT-PCR (**Figure 3**). Different expression patterns of different members were observed under ABA, water deficit, salt and low temperature stress. In general, ABA treatment showed the weakest stress response among the four treatments. The transcripts of *TaSnRK2s* in subclass III were induced strongly, *TaSnRK2s* in subclass II induced weakly, whereas *TaSnRK2s* in subclass I were not activated by ABA treatment.

Water deficit, simulated by PEG treatment, was the most powerful stimulant for *TaSnRK2* genes. All *TaSnRK2* members were induced quickly by PEG treatment, responding within 1 h or less, much more rapidly than other stressors. The expression patterns of all *TaSnRK2s* members (except *TaSnRK2.7*) under PEG treatment had unique expression profiles, responding with a bimodal pattern showing high expression at 3 and 24 h, but decreased expression at 3, 6, and 12 h after treatment. For most of the constructs, expression increased again at 24–48 h but decreased by 72 h.

All the *TaSnRK2s* were activated by NaCl and cold treatment. Among them, *TaSnRK2.10* and *TaSnRK2s* in subclass I responded to salt stress within 1 h. The transcriptional maxima of other *TaSnRK2s* were variable; constructs in subclass II as well as *TaSnRK2.8* and *9*, showed high levels of transcription at 48 h under salt stress. Low temperature also resulted in higher levels of *TaSnRK2* transcription. Transcription generally increased over the time course, with maximal levels occurring at 6 h or later.

### Physical Interaction between SnRK2 and PP2C

**FIGURE 4:**
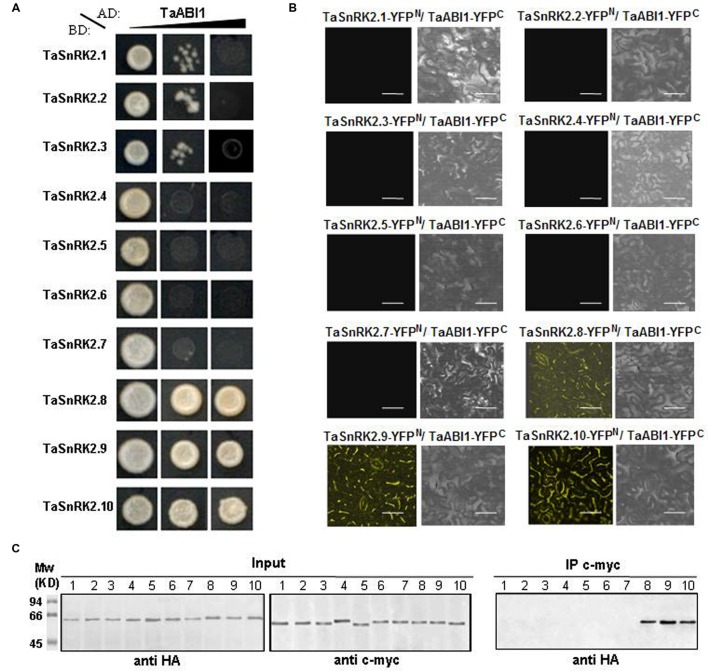
**Physical interactions among TaABI1 and TaSnRK2s.** **(A)** Yeast two-hybrid analysis of GAL4AD:TaABI1 and GAL4BD:TaSnRK2s were transformed to yeast cells as indicated. Black slopes indicate screening stringency of SD media supplemented with: left, -LW; center, -LWH + 10 mM 3-AT; right, -LWHA + 30 mM 3-AT. **(B)** BiFC image of TaABI1 and TaSnRK2s. BiFC analyses were performed in epidermal cells of tobacco leaves. Left, YFP fluorescence image; right, bright field. Bars = 50 μm. **(C)** The results of the BiFC experiments were confirmed by co-immunoprecipitation. The expression of the YFPN and YFPC fusion proteins in tobacco leaves was analyzed by immunoblotting using anti-c-myc and anti-HA antibodies. Proteins immunoprecipitated by the anti-c-myc antibody were analyzed by immunoblotting using anti-HA antibodies. Number 1–10 indicated TaSnRK2.n-YFP^N^/TaABI1-YFP^C^.

A typical group A PP2Cs, *TaABI1*, was cloned in a previous study (Nakamura et al., 2007). To better understand the biochemical relation between PP2C and each TaSnRK2 member in ABA signaling, we tested their interaction by Y2H, BiFC assay, and co-immunoprecipitation (**Figures 4A–C**, respectively). As shown in **Figure 4A**, *TaABI1* strongly interacted with TaSnRK2 subclass III members, while showing limited or no interaction with subclasses I and II members. To confirm these interactions *in vivo*, the two classes of proteins were co-expressed in tobacco cells using the BiFC assay. In the BiFC assay, interacting partners bring the N-terminal and C-terminal components of YFP together, resulting in YFP fluorescence. Each *TaSnRK2* N-term fusion was co-transfected with the C-term *TaABI1* fusion construct. Confirming our Y2H results, the C-term-*TaABI1* construct complemented subclass III N-term constructs to give strong YFP fluorescence in tobacco cells (**Figure 4B**). We further confirmed this result with co-immunoprecipitation of TaABI1:c-myc fusions with subclass III TaSnRK2s:HA fusions (**Figure 4C**). Together, these results indicate that TaABI1 interacted strongly with subclass III TaSnRK2s, whereas there was no evidence for interaction with subclasses I and II TaSnRK2s.

## Discussion

Plant stress resistance is achieved by complex networks of signal transduction via both ABA-dependent and ABA-independent resistance mechanisms. It was reported that the SnRK2 family of *Arabidopsis*, rice and maize have evolved specifically to respond to various types of stress and that individual members have acquired distinct regulatory properties, including ABA responsiveness (Kobayashi et al., 2004; Boudsocq et al., 2007; Huai et al., 2008). Here, 10 SnRK2 members in wheat were cloned and characterized. Gene structure and phylogenetic analysis showed similarity between SnRK2 members of wheat to counterparts in *Arabidopsis*, maize and rice, implying the SnRK2 kinase evolved before the divergence of dicots and monocots.

TaSnRK2s contain two typical domains, the conserved N-terminal catalytic domain and the somewhat variable C-terminal domain. Compelling evidence showed that the C-terminal domain plays a role in the activation of the kinase by participation in protein–protein interactions, mainly involved in ABA responsiveness and signal transduction (Kobayashi et al., 2005; Riichiro et al., 2006; Florina et al., 2009). Conserved motif analysis indicated that seven motifs were unevenly distributed in the C-terminus of TaSnRK2s. Among them, motifs 1 and 3 existed almost all the members, motif 2 was present in subclasses II and III, motif 6 was unique to subclass I, and motifs 4 and 5 were unique to subclass III. This is in agreement with findings in the maize SnRK2 family, suggesting that: (1) motifs 1 and 3 formed before the differentiation of three classes and were essential for the regulatory function of the C-terminus; (2) gene members classified in same group have same motifs, perhaps reflecting similar functions; (3) motifs 4 and 5 might participate in the ABA response according to the specific class (Huai et al., 2008). Further studies are required on the motif-exchange experiment using protein interaction assay.

Transcription patterns can indicate a gene’s involvement in functional or differential events. Numerous studies have demonstrated that SnRK2 is involved in multiple abiotic stress responses. In this study, the expression of *TaSnRK2s* was induced by water deficit, salt and cold, and individual members have acquired distinct regulatory properties. The transcripts of subclass III *TaSnRK2s* were strongly induced by ABA, subclass II *TaSnRK2s* responded to ABA weakly and *TaSnRK2s* in subclass I were not activated by ABA treatment. These results suggest that *TaSnRK2s* are involved in various abiotic stress responses via different mechanisms. Most *TaSnRK2s* were highly sensitive to osmotic stress because this stressor strongly induced transcription within an hour of exposure and generally caused the highest levels of transcription compared to the other stressors. Evidence from cultured plant cells support these findings, showing extremely early activation of *NtOSAK* and *AtSRK2C*, implied that *SnRK2s* may be induced immediately by osmotic stress (Kelner et al., 2004; Umezawa et al., 2004).

Analyzing the *cis* elements in the promoters of *TaSnRK2s* showed that their promoters are differentially regulated and carry a large number of stress-related *cis* elements. However, it was found that genes induced by a particular stressor do not all have corresponding *cis* elements in their promoters. For instance, only two ABREs were present in *TaSnRK2.8* promoter, although it was strongly induced by ABA. There were 13 ABREs in *TaSnRK2.6*, while it was not activated by ABA. These findings indicate that there are likely other unrecognized stress-related *cis* elements and/or unknown mechanisms involved in the regulation of these genes.

Increasing evidence show that SnRK2 genes play important roles in abiotic stress responses in plants via ABA dependent or independent signaling pathways. The subclass III SnRK2 kinases are the center of the ABA signaling network through interaction between SnRK2 and PP2C (Yoshida et al., 2006; Fujita et al., 2009; Umezawa et al., 2009). These findings were supported by the Y2H and BiFC assays reported in this study. Furthermore, subclasses I and II TaSnRK2s failed to interact with group A PP2Cs. However, subclass II *TaSnRK2s* were weakly activated by ABA in the expression pattern analysis. These results suggested that (1) subclass I *TaSnRK2s* respond to various abiotic stresses in an ABA-independent manner; (2) subclass II *TaSnRK2s* might participate in ABA independent signaling involved in cross-communication between ABA-dependent and ABA-independent signaling networks. Further studies are required on the relationship between SnRK2 kinases and the ABA independent signal transduction pathway; in addition, a better understanding of the biochemical relationships between PP2C and SnRK2 will elucidate the molecular mechanisms of SnRK2 and adaptive cellular mechanisms in stressed plants.

This study primarily concerned the characterization of wheat *SnRK2* genes involved in abiotic-stressed responses. Further comprehensive investigations to demonstrate the biochemical relation between PP2C and each SnRK2 were studied. The results of these studies enable us to dissect the actual molecular mechanisms of each *SnRK2s* in abiotic stress response.

## Author Contributions

HZ and WL performed the experiments and participated to the data analysis. XM performed the qRT-PCR experiments, RJ projected design and supervision. HJ analyzed the data and revised the manuscript.

## Conflict of Interest Statement

The authors declare that the research was conducted in the absence of any commercial or financial relationships that could be construed as a potential conflict of interest.

## Footnotes

1 http://meme.sdsc.edu/meme/meme.html

2 http://www.ebi.ac.uk/ena/search/

3 http://www.nias.affrc.go.jp/eng/database2/index.html
