# Supplementary material for: Differential Activation of the Wheat SnRK2 Family by Abiotic Stresses
Source: Front Plant Sci. 2016 Mar 31;7:420. doi: 10.3389/fpls.2016.00420 (PMC4814551; doi:10.3389/fpls.2016.00420)
Supplement: Supplementary file 1 [file Table_1.DOC]

Table S1. Primers used in this study.

| **Name** | **Sequence (5' to 3')** | **Description and purpose** |
| --- | --- | --- |
| *TaSnRK2.1* | GGAGGATGGATCGGTACGAGG | Forward primer for full-length |
|  | ACACGGTAAACCATCAAGAGAACT | Reverse primer for full-length |
|  | CGCAGAAACCTGGCGATAAC | Forward primer for real time Q-PCR |
|  | GCACACGAAATCCCCGCT | Reverse primer for real time Q-PCR |
|  | GAGAGAATTCGATGGATCGGTACGAGGTGGTG | Forward primer for cloning gene in pGBKT7 |
|  | GAGAGGATTCGCACACGAAATCCCCGCT | Reverse primer for cloning gene in pGBKT7 |
|  | AGAGACTAGTGATGGATCGGTACGAGGTGGTG | Forward primer for cloning gene in pUC-SPYNE |
|  | GAGACCCGGGGCACACGAAATCCCCGCT | Reverse primer for cloning gene in pUC-SPYCE |
| *TaSnRK2.2* | CTGTCCACTCCACTCGCCACT | Forward primer for full-length |
|  | ACGACACACCAGAAGGATGAACAC | Reverse primer for full-length |
|  | GAGGACAGCGGCGACTTTG | Forward primer for real time Q-PCR |
|  | AGTTGCCCGAGCCTATGTCC | Reverse primer for real time Q-PCR |
|  | GAGAGAATTCGATGGAGCGGTACGAGGTGATC | Forward primer for cloning gene in pGBKT7 |
|  | GAGAGGTCTCGGATCCGCACACAAAGTCGCCGCT | Reverse primer for cloning gene in pGBKT7 |
|  | AGAGACTAGTGCACACAAAGTCGCCGCT | Forward primer for cloning gene in pUC-SPYNE |
|  | GAGAGGTCTCGGATCCGCACACAAAGTCGCCGCT | Reverse primer for cloning gene in pUC-SPYCE |
| *TaSnRK2.3* | GAGTTGGAGCCGCCCCTTG | Forward primer for full-length |
|  | CAGACAGACCGTATGAACTGCGAT | Reverse primer for full-length |
|  | GGAGCCGCCCCTTGAAAT | Forward primer for real time Q-PCR |
|  | GCCACCCCGAAGTTGCC | Reverse primer for real time Q-PCR |
|  | TCGAGAATTCGATGGAGGAGAGGTACGAGGCG | Forward primer for cloning gene in pGBKT7 |
|  | GAGACCCGGGGTAGGTCTCCCCCTCGGCTC | Reverse primer for cloning gene in pGBKT7 |
|  | GAGATCTAGAATGGAGGAGAGGTACGAGGCG | Forward primer for cloning gene in pUC-SPYNE |
|  | GAGACCCGGGGTAGGTCTCCCCCTCGGCTC | Reverse primer for cloning gene in pUC-SPYCE |
| *TaSnRK2.4* | CCAACCTACCGACCCAACGAAC | Forward primer for full-length |
|  | GCAGAAGTTCCACGATAGGCC | Reverse primer for full-length |
|  | GGTTCATGCAAGCGGAGAGC | Forward primer for real time Q-PCR |
|  | CAGCGAGCGGTGGTTGATG | Reverse primer for real time Q-PCR |
|  | TCGAGAATTCGATGGAGAAGTACGAGGCGGT | Forward primer for cloning gene in pGBKT7 |
|  | GAGAGTCGACCGAGCTCATGCGGAGCTCT | Reverse primer for cloning gene in pGBKT7 |
|  | GAGAGGATTCCGAGCTCATGCGGAGCTCT | Forward primer for cloning gene in pUC-SPYNE |
|  | GAGACCCGGGCGAGCTCATGCGGAGCTCT | Reverse primer for cloning gene in pUC-SPYCE |
| *TaSnRK2.5* | GAGAGAGCATGGAGAAGTACGAGC | Forward primer for full-length |
|  | ATTTGGAGCTTGCTCATGCCG | Reverse primer for full-length |
|  | GGTCGGGCAACTTCGGG | Forward primer for real time Q-PCR |
|  | GGTGCGTGGGCGTGAGC | Reverse primer for real time Q-PCR |
|  | TCGAGAATTCGATGCGCAACCGGGACACG | Forward primer for cloning gene in pGBKT7 |
|  | GAGAGGATCCCACGCTCTGCGCCGAGTAG | Reverse primer for cloning gene in pGBKT7 |
|  | AGAGACTAGTATGCGCAACCGGGACACG | Forward primer for cloning gene in pUC-SPYNE |
|  | GAGAGGATCCCACGCTCTGCGCCGAGTAG | Reverse primer for cloning gene in pUC-SPYCE |
| *TaSnRK2.6* | GCGCTCGGCTAGGCCCCGGGATCC | Forward primer for full-length |
|  | GTCTGTGGCCTTCTCGCCAGTG | Reverse primer for full-length |
|  | GGAGGTGCTCTCCCGCCGGGAATAC | Forward primer for real time Q-PCR |
|  | TGGGATACGTGGACGTACTCCGGTA | Reverse primer for real time Q-PCR |
|  | TCGAGAATTCCGGGATCCATGGAGAGGTACG | Forward primer for cloning gene in pGBKT7 |
|  | GAGATCTAGAGCTGATGTGGAACTCACCGCTG | Reverse primer for cloning gene in pGBKT7 |
|  | GAGATCTAGACGGGATCCATGGAGAGGTACG | Forward primer for cloning gene in pUC-SPYNE |
|  | AGAGACTAGTGCTGATGTGGAACTCACCGCTG | Reverse primer for cloning gene in pUC-SPYCE |
| *TaSnRK2.7* | CCCAATCTTCGCCTCTGCC | Forward primer for full-length |
|  | TTTATCCCCGGTCTGTGGCC | Reverse primer for full-length |
|  | CGGGGAGAAGATAGACGAGAATG | Forward primer for real time Q-PCR |
|  | CTCAAAAAGCTCACCACCAGATG | Reverse primer for real time Q-PCR |
|  | TCGAGAATTCGCCGGGATCCATGGAGAGGTA | Forward primer for cloning gene in pGBKT7 |
|  | GAGATCTAGAGCTGATGTGGAACTCGCCG | Reverse primer for cloning gene in pGBKT7 |
|  | GAGATCTAGACCGGGATCCATGGAGAGGTA | Forward primer for cloning gene in pUC-SPYNE |
|  | CCGCTCGAGGCTGATGTGGAACTCGCCG | Reverse primer for cloning gene in pUC-SPYCE |
| *TaSnRK2.8* | GGGGAAACCGAGCCCTATC | Forward primer for full-length |
|  | CAAGTTCAGTCACAGGTTCACACATTA | Reverse primer for full-length |
|  | CGGGGAGAAGATAGACGAGAATG | Forward primer for real time Q-PCR |
|  | CTCAAAAAGCTCACCACCAGATG | Reverse primer for real time Q-PCR |
|  | TCGAGAATTCGTCGCCGCCGGCCAT | Forward primer for cloning gene in pGBKT7 |
|  | GAGATCTAGACGCATACACGATCTCTCCACTG | Reverse primer for cloning gene in pGBKT7 |
|  | GAGATCTAGACAGTCGCCGCCGGCCA | Forward primer for cloning gene in pUC-SPYNE |
|  | CCGCTCGAGCGCATACACGATCTCTCCACTG | Reverse primer for cloning gene in pUC-SPYCE |
| *TaSnRK2.9* | GCGGCGCTGACGGTGG | Forward primer for full-length |
|  | CCAAAAGGCCAAACCGTTGTACTAC | Reverse primer for full-length |
|  | GTCAATCTTCTCCCCTCGCTC | Forward primer for real time Q-PCR |
|  | ATCATGCAGATCCTGGCGG | Reverse primer for real time Q-PCR |
|  | TCGAGAATTCGATGGACATGCCCATAATGCA | Forward primer for cloning gene in pGBKT7 |
|  | GAGATCTAGACATAGCATACACTATCTCCCCGCT | Reverse primer for cloning gene in pGBKT7 |
|  | GAGATATCGAGATGGACATGCCCATAATGCA | Forward primer for cloning gene in pUC-SPYNE |
|  | CCGCTCGAGCATAGCATACACTATCTCCCCGCT | Reverse primer for cloning gene in pUC-SPYCE |
| *TaSnRK2.10* | GCGAGCGAGAGAGATAAGGAGA | Forward primer for full-length |
|  | GTCCGAGTCCAGGTCATCCAT | Reverse primer for full-length |
|  | GGAGTCAGCTACTGCCACTCCATGC | Forward primer for real time Q-PCR |
|  | TATGCCGGAGTTCCAACAGTTGAC | Reverse primer for real time Q-PCR |
|  | TCGAGAATTCGGGGGCGATGGAGAGCGG | Forward primer for cloning gene in pGBKT7 |
|  | GAGATCTAGACGAGTCCAGGTCATCCATGTC | Reverse primer for cloning gene in pGBKT7 |
|  | AGAGACTAGTGGGGGCGATGGAGAGCGG | Forward primer for cloning gene in pUC-SPYNE |
|  | GAGATCTAGACGAGTCCAGGTCATCCATGTC | Reverse primer for cloning gene in pUC-SPYCE |
